# Ultrasound Treatment Increases Transfection Efficiency of Low Molecular Weight Chitosan in Fibroblasts but Not in KB Cells

**DOI:** 10.1371/journal.pone.0092076

**Published:** 2014-03-20

**Authors:** Ureporn Kedjarune-Leggat, Chanyapat Supaprutsakul, Wilaiwan Chotigeat

**Affiliations:** 1 Department of Oral Biology and Occlusion, Faculty of Dentistry, Prince of Songkla University, Hat-Yai, Songkhla, Thailand; 2 Department of Molecular Biotechnology and Bioinformatics, Faculty of Science, Prince of Songkla University, Hat-Yai, Songkhla, Thailand; University of Utah, United States of America

## Abstract

The aim of this study was to optimize transfection efficiency (TE) of the depolymerized low molecular weight (LW) chitosan with molecular weight (Mw) at 16 kDa and 54% degree of deacetylation (DDA) on three primary cells of fibroblast (F), dental pulp (P), and periodontal ligament (PDL). The effect of low frequency ultrasound treatment on the chitosan-DNA complexes prior transfection on TE was also evaluated. This LW chitosan required high N/P ratio (>34) to bind DNA completely. An N/P ratio above 56 tended to improve TE in most primary cells nearly at the level of Lipofectamine. Ultrasonication can reduce the aggregation and sizes of the chitosan-DNA microparticles. It increased TE of F cells at an N/P ratio above 34, which was higher than Lipofectamine. However, this ultrasound treatment caused loss of TE in KB cells. MTT assay of these chitosan-DNA complexes revealed no significant cytotoxicity to both KB and F cells. This LW chitosan has potential for further development into a safer alternative to gene delivery systems in various cells of interest; however the optimal conditions have to be adjusted, depending on each cell source.

## Introduction

An efficient gene delivery system for a wide range of cell types is central to the development of regenerative biomedicine and tissue engineering therapy. Non-viral vectors offer high safe and versatile alternative to viral counters have gain increasing interest. However, they have limited application due to the lack of transfection efficiency (TE) [1], especially with non-cancer or somatic cells.

Chitosan (poly[(-(1-4)-2-amino-2-deoxy-D-glucopyranose]), a nontoxic biodegradable biopolymer, has been widely used as a pharmaceutical excipient due to its biocompatibility, good bio-adhesiveness and general availability [2]. This biopolymer has been broadly studied for drug delivery [3] and as a scaffold for tissue engineering, as well as potential use as a non-viral vehicle for gene delivery [4], [5]. The high density of primary amine group makes chitosan bind spontaneously with negatively charged molecules, such as nucleotides, into nanoscale microparticles, which can more easily enter into cells via endocytosis [6], [7]. Thus, preserving its nanosize is achieved by carefully adjusting their formulation, preparation, modification, properties of the loading biomedical agent and the physicochemical characters of the carrier material. There are many studies concerning factors related to enhancing TE of this non-viral vehicle, including varying its molecular weight, degree of deacetylation (DDA), molar ratio between the amino positive charge of chitosan/negative charge of phosphate group from DNA (N/P), and the pH of transfection, as well as modification of its chemical structure (see review) [8], [9]. The TE of chitosan is also dependent on cell type [10], [11], [12]. However, most of the information has been gained from cancer or immortalized cell lines and limited studies have been performed on primary cell lines.

Some evidence has suggested that high molecular weight (Mw) chitosan may not be suitable for use as a transfection vehicle under normal physiological conditions due to aggregation, low solubility, high viscosity and slow dissociation or degradation [13]. Low Mw chitosan [14], [15] and its modifications [16], [17] have been successfully used for transfection of both DNA and siRNA [18]. This was supported by our previous study of low Mw chitosan of 16 kDa, which showed the potential for transfection *in vitro* [19]. One problem of chitosan-DNA nanoparticles that formed from low Mw chitosan has been the aggregation of the particles [20] before transfection, which enlarges the complexes’ size and may interfere with the transfection.

Ultrasound or sonication has been used efficiently to enhance transfection in animal cells and tissues [21], [22], [23]. It can disaggregate and reduce the size and polydispersity of chitosan nanoparticles, but high intensity of ultrasound can also damage the chitosan nanoparticles [24].

The aim of this study was to optimize TE of the depolymerized low molecular weight (LW) chitosan on some primary cell lines. The investigation of the effect of using low frequency ultrasound treatment to the chitosan-DNA complexes prior to transfection on transgene expression in fibroblasts, as a primary cell line, compared with KB, as a cancer cell line, was also included.

## Materials and Methods

### Materials

Chitosan (Mw ∼470 kDa, DDA ∼80%) used in this study was obtained from Fluka (Japan). BCA assay kit and dialysis membranes with molecular weight cut off at 3.5 kDa were sourced from Pierce chemical (Rockford, IL, USA). Lipofectamine 2000 reagent was purchased from (Invitrogen Corporation, Singapore). The Steady-GloTM Luciferase Assay kit was obtained from Promega (Madison, USA). All culture media were purchased from Gibco BRL (Invitrogen Corporation, USA). All other chemicals were of the highest grade commercially available either from Merck (Darmstadt, Germany) or Sigma-Aldrich (St. Louis, MO, USA).

### Cell Culture

#### Ethics statement

Three human primary cell lines, including human oral fibroblast cells (F), dental pulp cells (P) and periodontal ligament cells (PDL), were used in this study. Ethical approval was obtained from the ethics committee of the Faculty of Dentistry, Prince of Songkla University, Thailand (No. 0521.1.03/864) and donor’s written informed consents were given. In the case of a donor aged under 18 years, the consent in the written form was also given by the guardians.

Human epidermoid carcinoma cells (KB) were obtained from American Type Culture Collection (ATCC number CCL-17, Manassas, USA). Primary gingival fibroblast and primary periodontal ligament cells were cultured using direct explant technique [25]. The gingival tissue was obtained from four patients undergoing dental surgery for crown lengthening. F cells of passages between 4 and 20 were used. F cells clones were designated 122F (aged 16 yrs.; female), 124F (aged 52 yrs.; male) 128F (aged 56 yrs.; female), and 134F (aged 40 yrs.; male). The primary culture of PDL cells were obtained from 2 healthy human subjects. PDL tissues were scrapped from the third molar teeth extracted for orthodontic indications. PDL cells clones were designated 1PDL (aged 19 yrs.; female), and 3PDL (aged 18 yrs., male). All KB, F and PDL cells were grown at 37°C with 5% CO_2_ in DMEM supplemented with 10% fetal calf serum and 100 units/ml penicillin, 100 μg/ml streptomycin and 1% amphotericin B. The pulp tissues were taken from sound third molar teeth of 4 patients. P cells clones were designated 2P (aged 19 yrs.; female), 3P (aged 18 yrs.; female), 4P (aged 17 yrs.; male), and 5P (aged 18 yrs.; female). Primary culture of P cells was performed using an enzymatic method. Briefly, pulp tissue was minced into pieces and digested in a solution of 3 mg/mL of collagenase Type I and 4 mg/mL of dispase for 1 h at 37°C. After centrifugation, cells were cultured in alpha modified Eagle’s medium (αMEM), supplemented with 10% FCS, 100 μM L-ascorbic acid 2-phosphate (Sigma-Aldrich, MO, USA) 2 mM L-glutamate, 100 units/mL penicillin, and 100 μg/mL streptomycin, and incubated at 37°C with 5% CO_2_. P and PDL cells used in this study were from the third to fifth passages.

### Preparation of Plasmid DNA

The plasmids pGL4.13-Luc (Promega, USA) with 4.641 kb cDNA encoding for luciferase from the *Photinus pyralis* driven by an SV40 promoter were used for transfection. They were propagated in *E. coli* Top 10 competent cells and purified by precipitation method. The purified pDNA was then resolubilized in distilled water and its concentration/purity determined by UV spectrophotometry at 260 and 280 nm and by electrophoresis, respectively.

### Preparation and Characterization of Depolymerized Low Molecular Weight Chitosan

The depolymerized low molecular weight (LW) chitosan used in this study has a Mw of 16 kDa, Mn of 6.3 and 54% of DDA. It was the product of depolymerization reaction of the commercial chitosan (Mw ∼470 kDa, DDA ∼80%) with sodium nitrite. [19], [26]. Briefly, 2 g of chitosan was dissolved in 100 mL of acetic acid (6% v/v) under magnetic stirring at room temperature. Then, 80 mg of sodium nitrite in 10 mL of water was added. After 1 h of incubation, the depolymerized chitosan was precipitated by raising the pH to 9 with 4 M NaOH. The resulting precipitate was filtered and further washed thoroughly with cold acetone. The residue was then dissolved in 100 mL of 0.1 M acetic acid and dialyzed against water before concentrated partially under vacuum. The samples was lyophilized at 80°C and 0.01 mbar (Christ Alpha 2–4, Osterode am Harz, Germany) prior to characterization and used for further experimentation. The Mn and Mw of LW chitosan product was determined by gel permeation chromatogram (GPC) (Waters 600E, water). It’s DDA of free amine groups (−NH_2_) was also characterized by NMR spectrophotometry.

### Preparation of the Chitosan -DNA Complexes with and without Ultrasound Treatment

The chitosan-DNA complexes formulated with LW chitosan at N/P ratios of 3, 7, 34, 56, 68, and 81were prepared by a complex coacervation method (Corsi et al., 2003, Mao et al., 2001). Briefly, 0.02% chitosan were prepared in 5 mM of sodium acetate buffer at pH 5.5 and 100 μg/mL of DNA in 50 mM of sodium sulfate solutions were separately heated at 55°C for 30 min. The maximum final volume/reaction was limited to 500 μL. The mixtures were intensively vortexed for 2 min and left for 15 min to form microparticles at room temperature. Then, the chitosan/DNA complexes groups that were assigned for ultrasound treatment were treated in an ultrasound water bath (Cavitator Ultrasonic Cleanser, Mettler Electronic Corp., Anaheim, CA) at 67 kHz for 1 min before undergoing immediate transfection.

### Characterization of Chitosan -DNA Complexes

#### 1. Agarose gel electrophiresis

The DNA binding ability of chitosan and plasmid DNA were evaluated by electrophoretic mobility of the complexes of various ratios of chitosan to plasmid DNA. The complexes solutions containing 0.5 μg of DNA at various N/P ratios were loaded into individual wells of 1.0% agarose gel in 1×Tris-boric acid-EDTA buffer with 0.5 μg/mL ethidium bromide and electrophoresis was performed at 100 V for 45 min. The resulting DNA migration pattern was examined under UV irradiation.

#### 2. Particle size and zeta potential of the chitosan -DNA complexes

Samples of either with ultrasound or without ultrasound treatment of chitosan-DNA microparticles with N/P ratio of 3, 7, 34, 56, 68, and 81 were used to determined their size and zeta potential with a ZetaPALS system (Brookhaven Instrument Corp., Holtsville, NY, USA). The complexes were in the mixture solution of 5 mM of acetate buffer pH 5.5 and 50 mM sodium sulfate at a ratio of 1∶1. Particle size was analyzed at a scattering angle 90° via dynamic light scattering at 25°C. The measurement of zeta potential was utilized by phase analysis light scattering to detect electrophoretic mobility. Each complex was measured repeatedly 10 times.

### *In vitro* Transfection

F, P and PDL cells were seeded in a 24-well culture plate at a density of 7×10^4^ cells/well in 1 mL of DMEM containing 10% FBS, supplemented with 100 units/ml penicillin, 100 μg/mL streptomycin and incubated for 24 h at 37°C in 5% CO_2_. At the time of transfection, the medium was aspirated and cells were washed once with phosphate buffered saline (PBS, pH 7.4). The amount of chitosan-DNA microparticles equivalent to 5 μg DNA was added to each well and incubated for 24 h in culture media (DMEM with 10% FBS) at pH 6.9 without addition of antibiotics. Cells transfected with naked plasmid DNA cells and pDNA transfected with Lipofectamine 2000 reagent acted as controls. Transfection with Lipofectamine 2000 reagent followed the manufacture’s protocol by using 1 μg of pDNA mixed with 2 μL of Lipofectamine 2000 reagent. After 24 h, cells were washed with PBS before replaced with 1 ml of fresh complete medium and incubated for another 24 h. All experiments were repeated independently at least three times.

The non-ultrasound treatment of the chitosan-DNA complexes were used to transfect primary cultures of F, P and PDL cells from different subjects at N/P ratios from 3–81. The complexes with ultrasound treatment were used to transfect KB and 128F cells.

### Luciferase Activity Assay

The luciferase assay was carried out according to manufacturer’s instruction (Promega, Madison, USA). Cells were harvested by removing the medium and then washed with PBS. Thereafter, 100 μL of 1× Glo lysis buffer was added (Promega, Madison, WI, USA). The cell lysate was centrifuged at 10,000 rpm for 3 min and the supernatant was collected. An aliquot of 40 μL of Steady-Glo luciferase Assay System (Promega) reagent was added just prior to measurement on a luminometer (PerkinElmer, Wellesley, MA, USA). TE was reported as the relative light units (RLU) normalized to the protein concentration in the cell extracts of transfected cell, which was measured by the BCA method (Pierce Biotechnology).

### Cytotoxicity Testing

The *in vitro* cytotoxicity test of the chitosan-DNA complexes formulated with chitosan-DNA complexes formed at N/P ratios of 56 and 68 and at pH 6.9 and 7.4 were evaluated using the 3-(4, 5-dimethylthiazol-2-yl)-2, 5-diphenyltetrazolium bromide (MTT) assay [27] by exposing the complexes with KB and F cells for 24 and 48 h. Briefly, cells were seeded at 6000 cells/well in 96-multiwell dish and cultured at 37°C with 5% CO_2_ in DMEM supplemented with 10% fetal calf serum and 100 units/mL penicillin, 100 μg/mL streptomycin and 1% amphotericin B. After 24 h, the chitosan-DNA complexes at different N/P ratios, as described above, were added in each well and incubated for 24 h and 48 h respectively. After that, the medium was removed, 200 μL of fresh medium containing 10 mM Hepes pH 7.4 was added each well, and 50 μL MTT solution (5 mg/mL in PBS) was then added to each well and incubated in the dark for 4 h at 37°C. The medium and MTT were then removed and 200 μL of DMSO and 25 μL of Sorensen’s glycine buffer (0.1 M glycine plus 0.1 M NaCl equilibrated to pH 10.5 with 0.1 M NaOH) was added. The optical density (OD) of formazan production was measured at 570 nm. The optical density (OD) values corrected for a blank (medium only) of the experimental groups were divided by the control and expressed as a percentage of the control, which represented the percentage of viable cells.

### Statistical Analysis

Statistical data analyses of size and zeta potential value using two-way ANOVA with Bonferroni post test applied for paired comparison analysis were performed. The data from *in vitro* transfection studies were evaluated using Kruskal-Wallis test. The comparisons of transfection efficiency between ultrasound and non-ultrasound treatment in each N/P ratio of KB and F cells were performed by Mann-Whitney U-test. *P* values below 0.05 were considered as statistically significant.

## Results

### Characterization of Chitosan-DNA Microparticles

#### 1. Agarose gel electrophoresis

**Figure 1:**
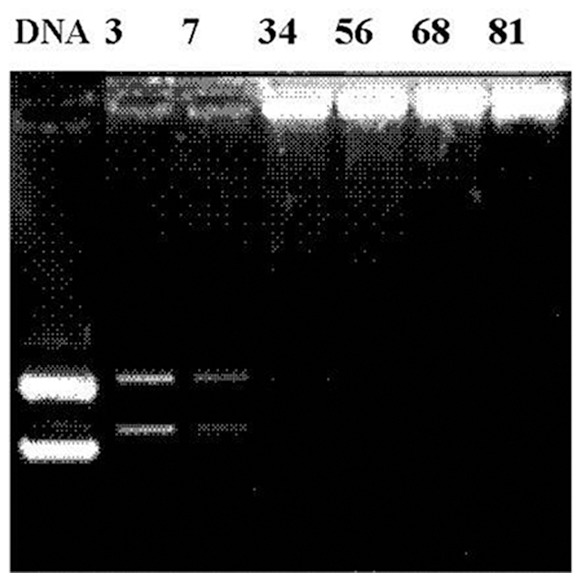
Electrophoretic mobility analyses of chitosan-DNA microparticles with different N/P ratios.

The optimal DNA condensation capacity based on chitosan carrier with various N/P ratios formulations was determined by varying the amount of chitosan and kept the DNA at 0.5 μg. As shown in Figure 1, the results clearly indicated that the electrophoretic mobility of all formulated chitosan-microparticles was retarded with the increasing amount of chitosan or higher N/P ratio. This LW chitosan could not bind DNA completely at low N/P ratios 3 and 7 and seemed to produce DNA trailing bands. Nevertheless, gradually decreasing the migration of uncondensed DNA into the gel was found with the increasing amount of chitosan. However, the complex formations were completely retarded at N/P ratios higher than 34.

#### 2. Particle size and zeta potential

**Figure 2:**
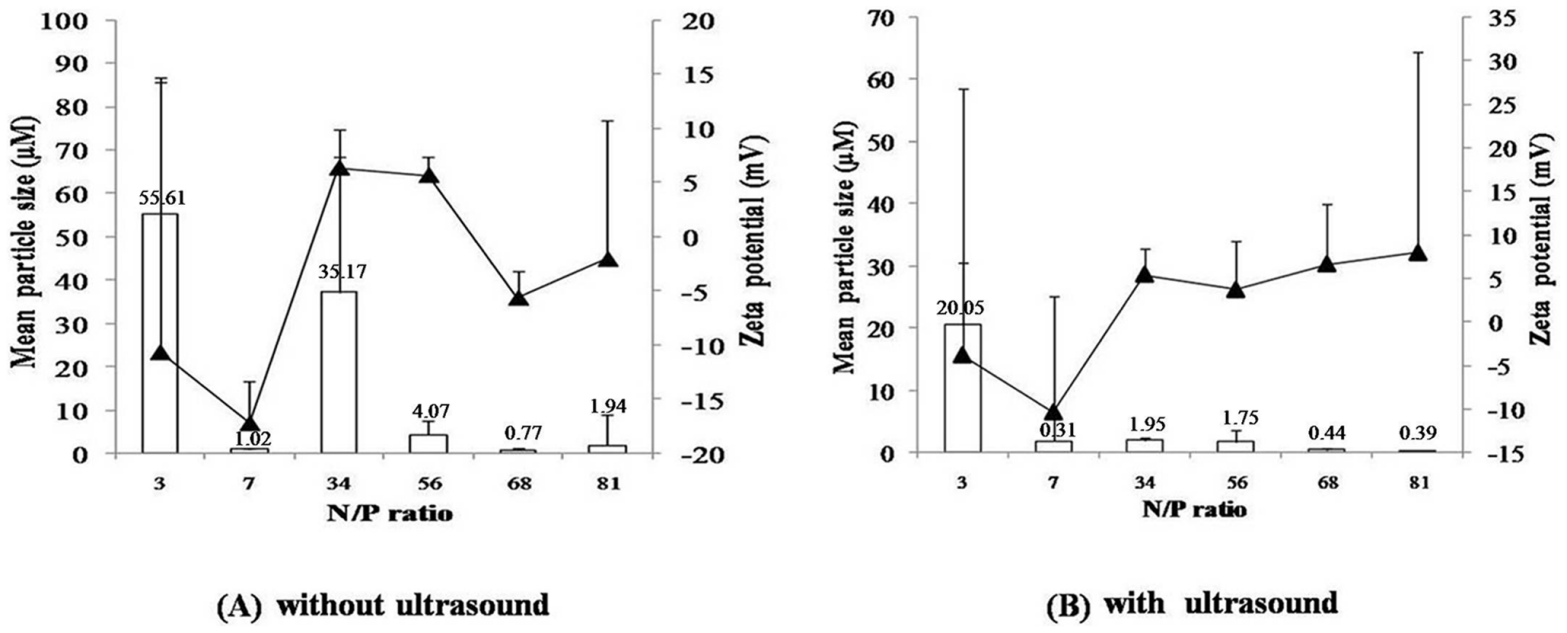
The mean size (□) and zeta potential (▴) of LW chitosan-DNA complexes at N/P ratios of 3–81. (A) without ultrasound treatment and (B) with ultrasound treatment.

The particle size and zeta potential of LW chitosan-DNA microparticles have been depicted in Figure 2. This illustrated how they clearly depended upon the N/P ratio and ultrasound treatment. Ultrasound treatment significantly reduced the mean size of the complexes (*P*<0.01, two-way ANOVA). In the non-ultrasound treatment groups, nearly all mean diameters were higher than 1 μm with high variations except at N/P ratio of 68, which gave a mean size of 776±338 nm. However, the ultrasound treatment groups gave lower complex sizes with the mean diameter of the complex size of N/P ratio 68 and 81 being 437±22 and 397±36 nm, respectively.

The zeta potential in the ultrasound treatment groups was also significantly higher than the non-ultrasound treatment groups (*P*<0.05, two-way ANOVA). In the non-ultrasound treatment groups, most of the zeta potential was negative, except in the N/P ratio at 34 being 6.4±3.6 and 56 being 5.7±1.7, while in the ultrasound treatment groups, most of the zeta potential was positive, except in N/P ratio 3 (−3.8±10.7) and 7 (−10.3±13.3).

### Transfection Efficiency (TE)

**Figure 3:**
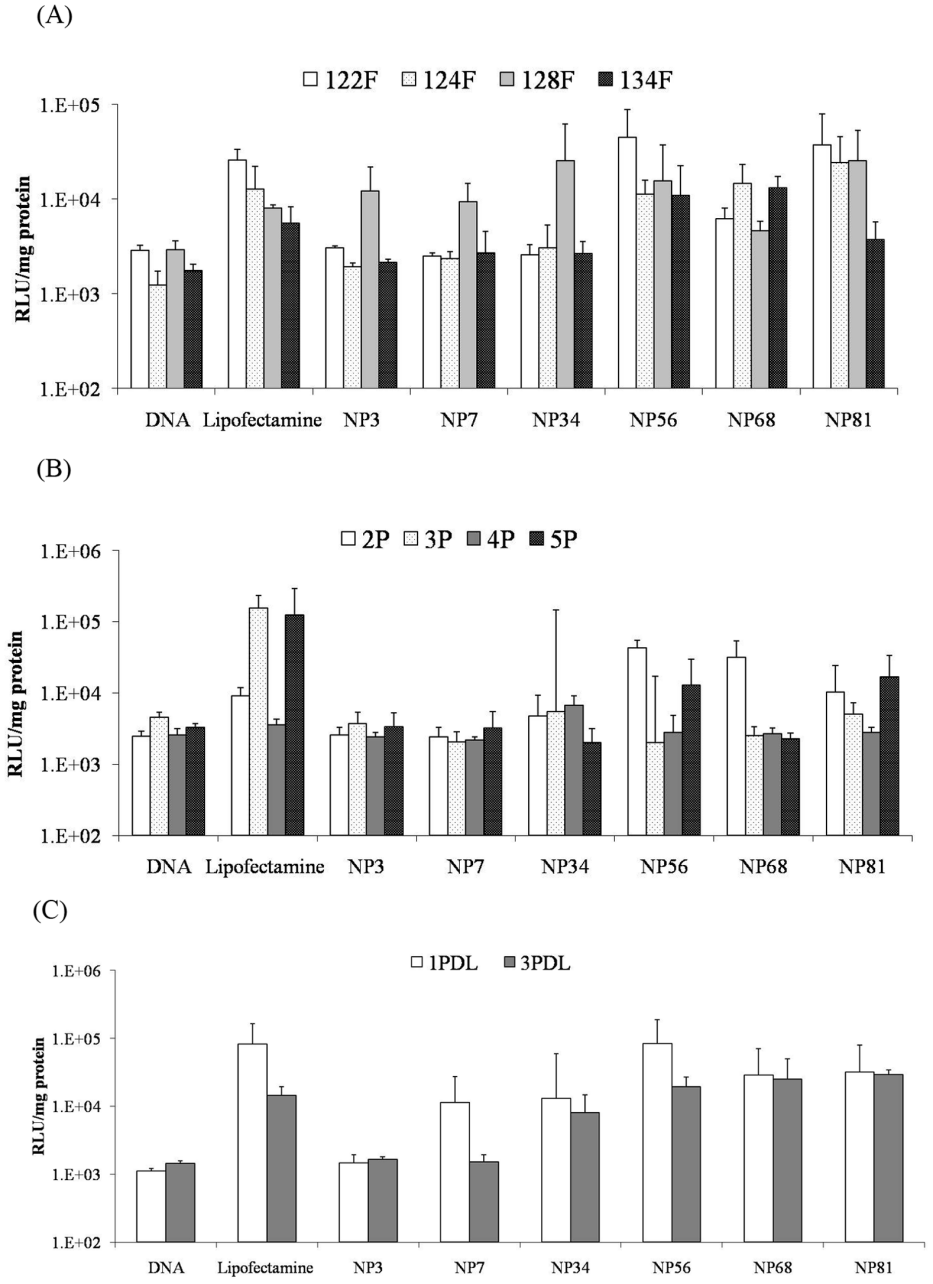
Luciferase expression in cells after transfection with chitosan-DNA complexes formed at different N/P ratios, (A), F cells, (B) P cells; (C) PDL cells.

The TE of F cells from four subjects was shown in Figure 3, which showed that Lipofectamine 2000 did not give the highest TE. The result demonstrated that F cells from different subjects also had different TE in responding to the same reagents. However, 122F had higher TE than other cell lines, while 134F had lower TE. The TE of chitosan-DNA complexes improved with the increased N/P ratio starting from N/P 34 to81. For example, 122F cells had high TE at N/P ratios 56 and 81, which were significantly higher than the naked DNA (*P*<0.05) and at the same level as Lipofectamine 2000. In 128F cells, both N/P ratio of 34 and 81 revealed the optimum TE about 0.25×10^5^ RLU/mg protein, which were about 3.18 fold higher than Lipofectamine 2000.

The TE of dental pulp cells (P) was low in both Lipofectamine 2000 and chitosan (see Figure 3B). In 2P cells, chitosan-DNA complexes at N/P ratios 56 and 68 gave significantly higher TE than DNA and Lipofectamine 2000 (*P*<0.05). However, in 3P and 5P, Lipofectamine 2000 gave the highest TE (*P*<0.05).

The TE of periodontal ligament fibroblast cells (PDL) was shown in Figure 3C. Both 3 PDL and 4PDL had higher TE than naked DNA at N/P ratios 56, 68 and 81, which was about the same level as Lipofectamine 2000.

**Figure 4:**
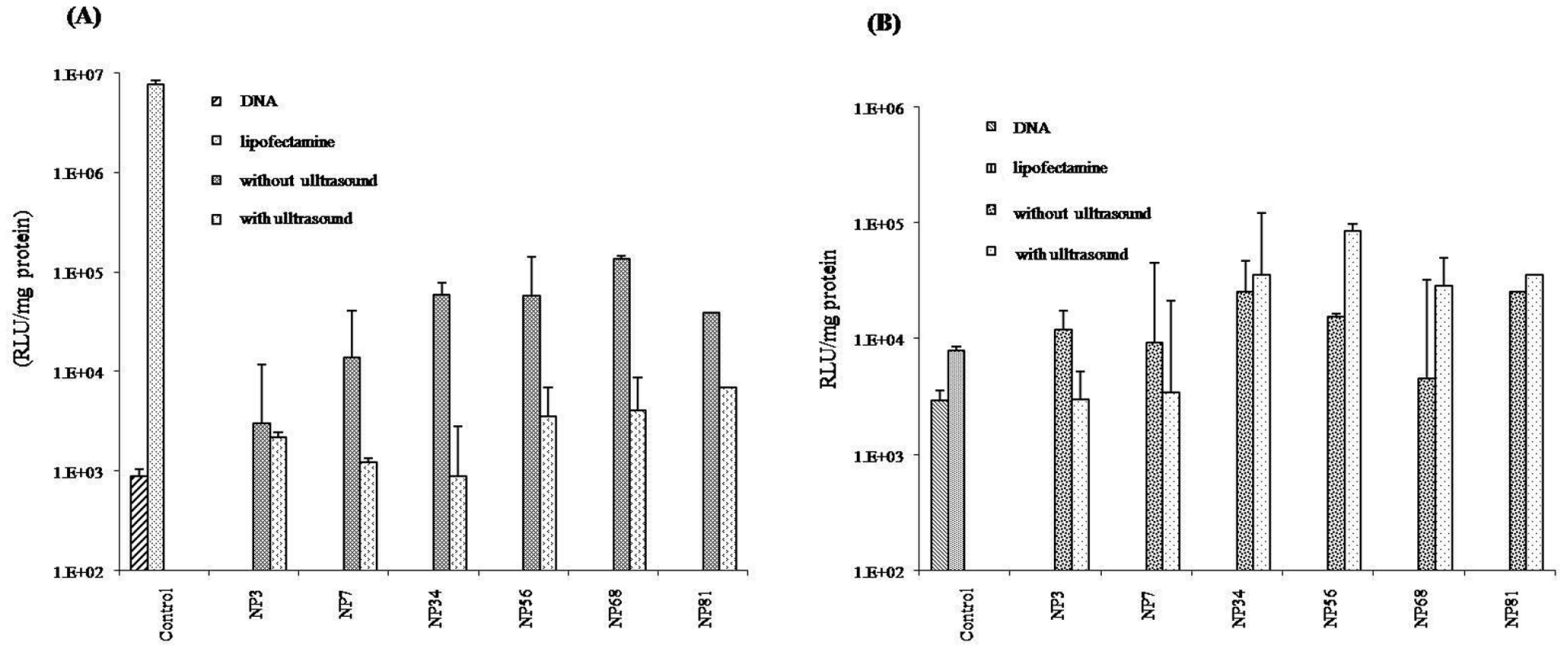
Luciferase expression in KB and F cells following treatment with and without ultrasound treatment of the chitosan-DNA complexes prior transfection. (A) KB cells; (B) F cells.

The effect of ultrasound treatment to the chitosan-DNA complexes on TE of KB and F cells has been shown in Figure 4 A and B. respectively. In F cells, the TE of the ultrasound treatment group was significantly higher than in the non-treatment group at N/P ratios 56 (*P*<0.05), 68 (*P*<0.01) and 81 (*P*<0.05). In particular, at N/P ratio 56, the TE of the ultrasound treatment group was highest (*P*<0.05) and about 10 fold higher than Lipofectamine 2000. However, the TE of the ultrasound treatment groups was significantly lower than the non- treatment groups in KB cells in all N/P ratios (*P*<0.05) (Figure 4 A).

### Cytotoxicity of Chitosan-DNA Complexes

**Figure 5:**
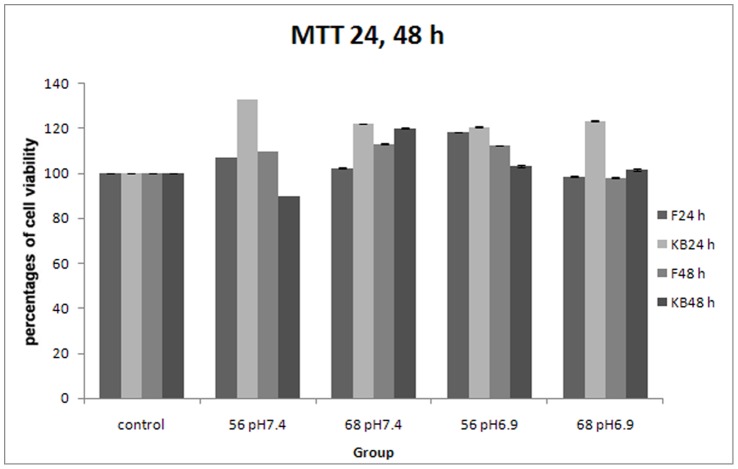
MTT assay of chitosan-DNA complexes on KB and F cells. Cells exposed to chitosan-DNA complexes formed at N/P ratios at 56 and 68 and pH 6.9 and 7.4 for 24 and 48 h, respectively.

The results of MTT assay evaluated the cytotoxicity of chitosan-DNA complexes formed at N/P ratio at 56 and 68 and pH 6.9 and 7.4 in KB and F cells has been shown in Figure 5. The percentages of viable cells of all experimental groups were higher than control groups (100%), which revealed no cytotoxic effect, except that the group of KB cells exposed to chitosan-DNA complexes at N/P ratio 56, pH 7.4 for 48 h had percentages of viable cells at 90%. However, the optical density (OD) of this group was not significantly different from its control group (*P*>0.05, t-test).

## Discussion

The results of this study indicated that the TE of chitosan-DNA complexes was strongly related to the N/P ratio, cell lines and ultrasound treatment. This LW chitosan required high N/P ratio (>34) to bind DNA completely. This may be because of the low molecular weight and also its low DDA, which may require more positive charges of the chitosan to stabilize the negative charges of DNA in the polyplexes. TE also improved at high N/P ratio. The excess of chitosan from high N/P ratio may enhance TE by facilitating intracellular lysosomal escape [28] after cellular internalization, if the main pathway was clathrin-dependent endocytosis [29]. But the internalization pathway may also depend on many factors, including type of cell line, complexes’ size, and cell physiology [12]. Nevertheless, if the amount of chitosan is too high, the chitosan-DNA complexes may retard the release of DNA after endocytosis which may explain why N/P 56 gave better TE than the N/P 68 and 81. This result confirmed the conclusion that too low N/P may yield unstable complexes while too high N/P may reduce transfection [30], [31]. Chitosan, which is efficient for DNA delivery, should bind DNA at the proper strength and can dissociate after internalization at the rate that provides enough time for lysosomal escape. The inefficient high molecular weight and high DDA chitosan resulted in too stable complexes and did not dissociate after 24 h of cell internalization [6].

Zeta potential was used as an indirect measurement of the particles surface charge density. Usually, high surface charge density of chitosan–DNA complexes determines the colloidal stability of non-sterically stabilized formulations [32]. The non-ultrasound treatment groups had low negative charge zeta potentials in both N/P 68 and 81, while at N/P 34 and 56 they had low positive charge zeta potentials. The low value of zeta potential may represent the instability of the colloid particles, which can easily aggregate. This may explain why the average particle sizes of the non-ultrasound treatment groups are higher than the ultrasound groups. The negative zeta potential in N/P 68 and 81 in non-ultrasound treatment groups may cause by DNA adsorption on the surface of the chitosan/DNA complexes, as chitosan-based DNA nanoparticles can be based on different mechanisms, including electrostatic interaction, encapsulation and adsorption [33].

DNA incorporated within the chitosan-DNA complexes plays a fundamental role in the efficiency of transfection. The plasmid concentration in this study was set at 5 μg/well for transfection, while chitosan concentration was varied for different N/P ratios. It was found that the TE will increase with plasmid concentration up to a critical point, and the transfection will then remain constant or decrease [33]. The increase of plasmid concentration from 0.5 to 2.5 μg will increase TE and a saturation in expression as a consequence of a further increase in the DNA concentration was found at 5 μg/well in epithelioma papulosum cyprini (EPC) cells [34]. In primary chondrocytes the plasmid dosage increased from 0 to 8 μg/well, the level of TE increased. When the plasmid dosage came up to 16 and 32 μg/well, the TE greatly decreased. This decrease may be the result of the aggregation of nanoparticles which reduced cell uptake [35].

Gingival fibroblast (F), periodontal fibroblast (PDL) and dental pulp (P) cells are all primary cell lines, which came from human oral tissue. They demonstrated different TE and even the same cell line type, but from different subjects, also had different TE. Cell physiology might be a critical factor in cellular internalization and transfection [36]. Further investigation is required to assist in providing an explanation.

From this study, ultrasound treatment can promote TE in fibroblast cell line, which may be the result from smaller size or reduced aggregation of the chitosan-DNA complexes. Conversely, this ultrasound treatment reduced the TE in KB cell line. The internalization of the complexes in both cell lines may be different. However, the result of this study suggested an easy method to improve TE in fibroblast cell line, even higher than Lipofectamine 2000, by using low power ultrasonic treatment of the chitosan-DNA complexes before transfection. Ultrasound has been previously used to mediate drug delivery (see review of Pua and Zhong [37]) and enhance gene delivery [23], [38], [39], [40] and these investigators believed that ultrasound can generate transient pore formation in the cell membrane known as sonoporation. However, this study applied low frequency and low power ultrasound treatment to the chitosan-DNA complexes in order to reduce the aggregation complexes, as being observed from the smaller sizes of the complexes. It may be interesting to further investigate whether chitosan can increase transfection efficiency in cells pre-treated with ultrasound. However, it is known that ultrasound can cause cell injury [41], so careful adjustment of its frequency and energy has to be seriously considered.

The low molecular weight chitosan with high DDA has been used in many studies and confirmed that it has low toxicity and high TE [15] and may be modified for site specific transfection [42], [43]. The problem of high DDA is the high strength of the DNA-chitosan binding, which may be slow in dissociation after internalization and particles tend to easily aggregate. This study used LW chitosan with low DDA, which also gave low toxicity, and, when used in the proper N/P ratio, it can give high TE, especially in primary cell lines, which normally has low TE.

## Conclusion

TE of this LW chitosan depended on cell line, N/P ratio and ultrasound treatment. This LW chitosan with low DDA required higher N/P ratios, above 34, to bind DNA completely and can give high TE in some primary cell lines. Ultrasound treatment of these chitosan-DNA microparticles prior to transfection can reduce their aggregation and sizes. It increased TE of F cells but decreased TE in KB cells. This LW chitosan has demonstrated some potential for further development towards a safer alternative to gene delivery systems in various human cells of potential interest.
